# Streptococcal Infections in Marine Mammals

**DOI:** 10.3390/microorganisms9020350

**Published:** 2021-02-10

**Authors:** Daniela Numberger, Ursula Siebert, Marcus Fulde, Peter Valentin-Weigand

**Affiliations:** 1 Institute for Microbiology, University of Veterinary Medicine Hannover, Foundation, Bischofsholer Damm 15, 30173 Hannover, Germany; Peter.Valentin@tiho-hannover.de; 2 Institute for Terrestrial and Aquatic Wildlife Research, University of Veterinary Medicine Hannover, Foundation, Werftstraße 6, 25761 Buesum, Germany; ursula.siebert@tiho-hannover.de; 3 Institute of Microbiology and Epizootics, Department of Veterinary Medicine, Freie Universität Berlin, Robert-von-Ostertag-Strasse 7-13, 14163 Berlin, Germany; Marcus.Fulde@fu-berlin.de

**Keywords:** streptococci, infectious diseases, marine mammals

## Abstract

Marine mammals are sentinels for the marine ecosystem and threatened by numerous factors including infectious diseases. One of the most frequently isolated bacteria are beta-hemolytic streptococci. However, knowledge on ecology and epidemiology of streptococcal species in marine mammals is very limited. This review summarizes published reports on streptococcal species, which have been detected in marine mammals. Furthermore, we discuss streptococcal transmission between and adaptation to their marine mammalian hosts. We conclude that streptococci colonize and/or infect marine mammals very frequently, but in many cases, streptococci isolated from marine mammals have not been further identified. How these bacteria disseminate and adapt to their specific niches can only be speculated due to the lack of respective research. Considering the relevance of pathogenic streptococci for marine mammals as part of the marine ecosystem, it seems that they have been neglected and should receive scientific interest in the future.

## 1. Introduction

The marine ecosystem is challenged by numerous factors such as anthropogenic pollution including wastewater [1,2], plastic [3], chemical [4,5], and noise pollution [6], fisheries and prey depletion [7,8], offshore-construction [4], shipping [9,10], harmful algal blooms [11,12], climate change, and acidification [13,14,15,16,17]. Marine mammals are considered sentinels for the marine ecosystem [18,19,20] and, thus, the assessment of their health status should be of global interest and importance. Cumulative effects caused by different anthropogenic activities (e.g., pollution of the environment with PCBs, PBDEs, microplastic) can have suppressive effects on the immune system of marine mammals and this might result in a higher susceptibility for infectious diseases [21,22,23,24]. There are a number of studies indicating a higher prevalence of or risk for infectious disease in correlation with those (anthropogenic induced) threatens. For instance, in a recent study, microplastic was found in each of 50 tested animals belonging to 10 different marine mammal species. Furthermore, animals that died due to infectious diseases showed a slightly higher amount of microplastic in the intestines [3]. Sanderson and Alexander [17] found that climate related factors such as sea surface temperature have a significant effect on the occurrence of infectious disease-induced mass mortality events. In another study, the risk for infectious disease mortality increased by 2% for each 1 mg/kg increase of polychlorinated biphenyls (PCBs) in the blubber of harbor porpoises [25].

Infectious diseases are one of the most frequent causes of death in marine mammals [26,27,28,29,30,31,32,33] and beta-hemolytic streptococci are frequently isolated and associated with disease [26,30,31,34,35,36,37]. The genus *Streptococcus* belongs to the family *Streptococcaceae*, the order *Lactobacillales*, the class *Bacilli,* and the phylum *Firmicutes*. Streptococci are non-motile, Gram-positive, catalase-negative, non-spore forming, and chemo-organotrophic with a fermentative metabolism [38,39]. They appear as ovoid cocci in pairs or chains and can be classified by their ability to cause different forms of hemolysis and their serologically active antigens according to the Lancefield classification scheme. Most of the currently described 79 streptococcal species are summarized in groups based on their 16S rRNA sequence, their pathogenic potential and specific characteristics. The *pyogenic* group includes human and animal pathogens, while the *anginosus*, *mitis* and *salivarius* groups also include commensals of the oral cavity and pharynx of humans, which, however, can also cause disease, such as endocarditis. Members of the *mutans* group colonize tooth surfaces and the *bovis* group contains members of the colon microflora [38,39,40,41].

Pathogenic streptococci can express a large arsenal of virulence(-associated) factors, such as exotoxins and enzymes, antiphagocytic hyaluronic acid containing capsule, host matrix protein binding surface proteins complement evasion factors, and, last but not least, antiphagocytic M- and M-like proteins [42,43,44].

However, the etiology and pathogenicity of streptococci to marine mammals are largely unknown. There is some debate as to whether streptococci are primary or opportunistic pathogens [34,45,46,47,48,49]. This discussion is further hampered by the lack of species identification in many reports. On the other hand, new species are regularly found in marine mammals such as *S. halichoeri* [50], *S. marimammalium* [51], and *S. phocae* [34]. 

Besides, some streptococcal species are known as zoonotic agents [52]. For instance, human meningitis was acquired as zoonoses from *S. equi* subsp. *zooepidemicus* after contact with domestic animals [53]. *S. suis*, a commensal and opportunistic pathogen of pigs, is also known to cause infections in humans [54,55]. *S. canis* and *S. iniae* are also zoonotic pathogens [56,57,58]. Thus, the ubiquitous distribution and zoonotic potential of streptococcal species represents a global health risk for animals and humans.

This review attempts to summarize what is currently known about streptococcal species detected in marine mammals and discusses important issues that deserve more attention in future research.

## 2. Streptococcal Findings in Marine Mammals

**Figure 1:**
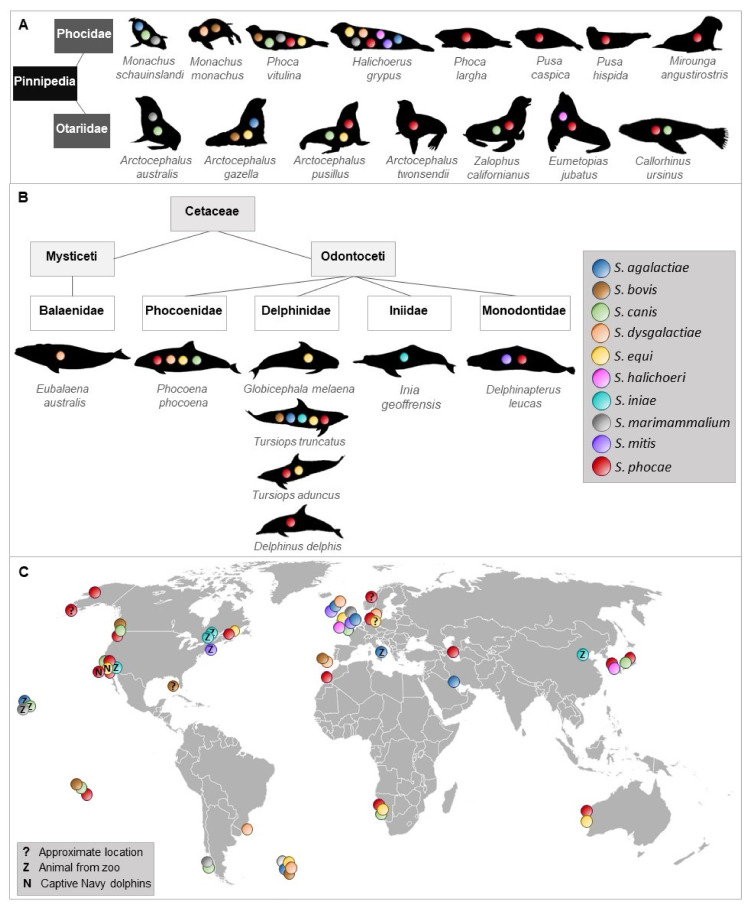
Occurrence of streptococcal species described in different marine mammals. Streptococcal species that have been isolated and identified at least twice in pinnipeds (**A**) and cetaceans (**B**). (**C**) shows a world map indicating location of streptococcal species detected in marine mammals.

To the best of our knowledge, 10 streptococcal species were isolated and identified more than once from 23 species of Pinnipedia and Cetaceae worldwide (Figure 1, Supplementary Table S1). 

### 2.1. Streptococcus agalactiae

*S. agalactiae*, also known as a bovine and human pathogen [59,60,61], was isolated from a wound and navel infection of grey seals (*Halichoerus grypus*) in North Rona in 1985 and 1986 [62] and from lesions of fight wounds, pneumonia, lymphadenitis as well as from lung and spleen samples of Antarctic fur seals (*Arctocephalus gazella*) from 1984–1987 on Bird Island, South Georgia [63]. Later, it was isolated from lesions and visceral organs (liver and lung) of a captive common bottlenose dolphin (*Tursiops truncatus*) that suffered from fatal necrotizing fasciitis and myositis [64]. One year later, the isolation of *S. agalactiae* from epaxial muscles of a wild stranded bottlenose dolphin was reported [65]. This strain caused 90% mortalities in tilapia in experimental infections and showed high similarity with strains associated with mullet kill in the concurrent Kuwait Bay. A mullet was found in the stomach of the dead dolphin, which might have served as a possible way of transmission. A study of human *S. agalactiae* strains from fish, seals, a dolphin, and a frog indicated zoonotic and anthroponotic hazard by causing severe disease in fish and compromising food security [66]. Between 2012 and 2014, *S. agalactiae* was isolated from a stranded grey seal on the British coast with ocular pathology [67]. In the Waikiki Aquarium, Honolulu, Haiwaii, *S. agalactiae* was isolated from two male healthy Hawaiian monk seals (*M. schauinslandi*) as part of their aerobic bacterial flora in the nasal cavity [68]. 

*S. agalactiae* is also known as serious fish pathogen [69,70,71]. In Brazil, high virulent strains were isolated from diseased Nile tilapia and transmission occurred by direct contact or through water [70]. Infection experiments confirmed the disease and revealed low LD_50_ for Nile tilapia. However, isolates from cattle did not cause any clinical signs in Nile tilapia and channel catfish indicating host specification and adaptation [72]. Human and bovine strains of *S. agalactiae* were able to cause disease in Nile tilapia, although there was no genetic relatedness of strains from fish, bovine, and human origin [73]. This suggests that the ability to cross host-specific barrier is not necessarily reflected by genetic linkage. Virulence gene profiling of *S. agalctiae* isolated from diseased tilapia in Thailand revealed a positive correlation of virulence genes content and pathogenicity [74]. Virulence genes for adhesion, invasion, and immune evasion were identified. Another study demonstrated that there were fish-specific genes or loci that were associated with disease in fish, while strains missing these regions were not able to cause morbidity in tilapia [75]. In addition, these fish-specific genes were mainly clustered in regions with signatures of mobile genetic elements and one fish-specific gene was found in the region, where the virulence genes *rib* or *bca* are in the human strain indicating genetic adaptation to the fish host. 

### 2.2. Streptococcus bovis

*S. bovis* has been isolated from the gastrointestinal tract and feces of cattle, sheep, goats [76], and dogs [77]. It has also been identified as human pathogen associated with endocarditis [78], meningitis [79], septic arthritis [80], bacteremia, and gastrointestinal disease [81]. Virulence factors associated with *S. bovis* infection were, for instance, extracellular proteins [82] and antigens [83]. *S. bovis* was detected in fur seals with pneumonia that was characterized by extensive polymorphonuclear infiltrations and necrosis or very widespread abscess formation and, frequently, by additionally fibrinous exudative pleurisy [63]. Together with *S. phocae* and *S. canis* it was also isolated from dead herpesvirus-positive harbor seal pups at the Smith Island, Washington [32]. A monk seal pup (*Monachus monachus*) found on the island Deserta Grande, Portugal died due to septicemia and *S. bovis* was isolated and considered as a potential causative agent [84]. In 2006, *S. bovis* isolation (together with *S. equisimilis/mitis*) from free-ranging bottlenose dolphins that were captured, sampled, and released in coastal Gulf of Mexico and Atlantic ocean waters was reported [85].

### 2.3. Streptococcus canis

*S. canis* was first isolated from cows with mastitis and from dogs with different pathological findings [86], but can also cause infections in humans [56,57,87]. It has also been isolated from minks [88], feral cats [89] and cats [90]. Its virulence and pathogenicity were confirmed by the presence of virulence genes such as for fibronectin-binding protein, M proteins, protective antigen, and streptolysin [91,92,93,94]. The M protein of *S. canis* has also a high-affinity immunoglobulin G binding activity, which is not species-specific and facilitate *S. canis* to interact with different hosts [95]. 

A beta-hemolytic *Streptococcus* sp., biochemically similar to *S. canis*, was cultured from pyogranulomatus lesion of the laryngeal cartilages and epiglottis of an adult harbor seal (*Phoca vitulina*) [27]. *S. canis* was also isolated from peritoneal effusion of a captive California sea lion (*Zalophus californianus*) of the US Navy’s marine mammal program [96] and from a California sea lion with bilateral corneal ulceration of the London Zoo, UK [97]. During an increased mortality among South American fur seal (*Arctocephalus australis*) pups on Guafo Island, Chile, South America, *S. canis* together with *S. marimammalium* were isolated and associated with moderate to marked, multifocal, mucopurulent bronchopneumonia [98]. In August 1994, *S. canis* (and also *S. phocae*, *S. equi* subsp. *zooepidemicus*) was isolated from spleen, liver, and kidney of Cape fur seals (*Arctocephalus pusillus pusillus*) at Cape Cross, Namibia that suffered from respiratory infections and abortions associated with starvation [99]. Seven cases of stranded harbor porpoises (*Phocoena phocoena*) in England and Wales between 1990 and 1996 had a *S. canis* septicemia, which was isolated from lungs with pulmonary abscesses and enlarged pulmonary associated lymph nodes [100]. *S. canis* (together with *S. phocae*) was cultured from blood and lung samples of a dead, stranded Northern fur seal (*Callorhinus ursinus*) with necrotizing and fibrinous pneumonia infiltrated by band neutrophils with intraluminal abscess of bronchi at the coast of Niigata, Japan [46]. In 2005, the isolation of *S. canis* from two dead harbor seal pups on Smith Island, Washington was reported [32,101]. One died from omphalophlebitis and the other from omphalitis with subsequent peritonitis. *S. canis* was also isolated from the oral cavity of a male, healthy Hawaiian monk seal of the Waikiki Aquarium, Honolulu, Hawaii [68].

### 2.4. Streptococcus dysgalactiae 

*S. dysgalactiae* subsp. *equisimilis*, previously known as *S. equisimilis* [102] and found in humans [103] and different animals such as dogs, cows, pigs, and horses [104], was isolated from Antarctic fur seal pups with septicemia and rhinitis in South Georgia, UK between 1979–1982 [105] and 1986 from a grey seal cow on North Rona, Scotland [62]. In the years 1988 and 1989, an increased number of harbor porpoise carcasses was observed in North and Baltic Seas [36]. Thirty-five isolates of beta-hemolytic streptococci were classified in Lancefield group L and identified as *S. dysgalactiae* subsp. *dysgalactiae*. In 1997, *S. dysgalactiae* and *S. dysgalactiae* subsp. *equisimilis* isolates were found in a dead, wild monk seal pup in association with a septicemia on the island Deserta Grande, Portugal [84]. Three isolates identified as *S. dysgalactiae* subsp. *dysgalactiae* were obtained from phocid seals (harbor and grey seals) stranded in the North and Baltic Seas between 1995 and 2000 [106]. Between 2005–2011, pathologic and microbiological findings of a southern right whale (*Eubalaena australis*) calf from Brazil indicated that beta-hemolytic *S. dysgalactiae* septicemia was responsible for the death of the animal [107]. 

### 2.5. Streptococcus equi

*S. equi* causes infections in horses [108] and was associated with canine infectious respiratory disease [109]. A systemic infection with *S. equi* in a horse handler has also been reported [110]. Further studies confirmed the zoonotic potential of *S. equi* [53,111]. In November 1978, a female North Atlantic pilot whale (*Globicephala melaena*) suffering from bronchopneumonia with lesions stranded on Metis Beach, Canada and *S. equi* (no further identification) was isolated from lung parenchyma, pharynx, and pericardial fluid [35]. A study from 1980 reported the isolation of *S. equi* subsp. *zooepidemicus* (previously *S. zooepidemicus*) from grey seals associated with mild, purulent pneumonia [49]. In 1994, it was isolated from the conjunctiva and trachea of two adult female Cape fur seals that had septicemic *S. phocae* in Namibia [99]. A total of 32 beta-hemolytic streptococcal isolates, collected during the phocine distemper outbreak in 2002 from 28 different harbor seals of the German North Sea, were identified as *S. equi* subsp. *zooepidemicus* [112]. Later, the same scientists isolated the same or at least very close related strains of *S. equi* subsp. *zooepidemicus* from grey seals and other harbor seals [113]. A retrospective study on 42 dead bottlenose dolphins from the US Navy Marine Mammal Program during 1980-2010 demonstrated an association of the isolation of *S. equi* subsp. *zooepidemicus* with pneumonia [114]. 16S rRNA sequences for *S. equi* (and *S. phocae*) were found in blow samples collected from four wild healthy Indo-Pacific bottlenose dolphins (*T. aduncus*) in Shark Bay (SB), Western Australia, in 2012 [115].

### 2.6. Streptococcus halichoeri

*S. halichoeri*, characterized as non-hemolytic and classified in Lancefield group B, was first isolated from dead grey seals in Iverness and Cornwall, UK [50] and few years later, in 2012, also from the kidney of a stranded, female Stellar sea lion (*Eumetopias jubatus*) in South Korea [116]. Also, in 2012, a severe case of a human infection with *S. halichoeri* was reported [117]. The patient had no contact to seals, but to fish, which could have been a possible transmission route. However, this was not tested. Another human infection was reported in 2018, where a man suffered from skin cellulitis due to *S. halichoeri* [118]. Shewmaker et al. [119] compared human and seal strains and concluded two subspecies *S. halichoeri* subsp. *halichoeri* for the seal isolates and *S. halichoeri* subsp. *hominis* for strains associated with human infections. The core genome of 20 *S. halichoeri* isolates from different hosts including dogs and minks contained 19 different streptococcal virulence factors, of which most were associated with adherence followed by proteases and toxins emphasizing its pathogenic potential [120,121].

### 2.7. Streptococcus iniae

*S. iniae* was described as new species in 1976, when it was first isolated from a captive Amazon freshwater dolphin (*Inia geoffrensis*) suffering from a dermatologic syndrome called “golf ball disease” in the Steinhart Aquarium, San Francisco, USA [122]. Further isolates were obtained from captive freshwater dolphins housed at the Niagara Falls Aquarium in New York, USA two years later [123], and in 1983 from a captive Amazon River dolphin at the Pittsburgh Zoo, USA that also developed the “golf ball disease” [124]. In 2015, a common dolphin died due to bacterial septicemia at Beijing aquarium, China, where *S. iniae* was isolated from hilar lymph nodes and pancreas of the dolphin [125].

*S. iniae* is also a serious fish pathogen [58,126]. Virulence mechanisms include a capsule with antiphagocytic function [127], the cytotoxin ß-hemolysin streptolysin S [128], an extracellular nuclease and s secreted nucleotidase that play an important role in immune evasion [129], a polysaccharide deacetylase involved in adherence, invasion, lysozyme resistance and survival in fish blood [130], and M-like protein [131]. Comparative genomics revealed genetic differences between strains from different hosts including *I. geoffrensis* and identified two plasticity zones that reflect adaptation to specific host environments [132]. Furthermore, the dolphin isolates differed from the fish and human isolates in lacking a capsule, forming denser and thicker biofilms, increased ability to withstand oxidative stress and were genetically highly divergent to the other isolates [133]. In addition, there were conserved mutation rates and mismatch/oxidized-guanine repair systems within phylogenetic clades, but significant differences between major phylogenetic lineages. Mutators might facilitate adaptation to novel hosts including immune escape. This indicates that *S. iniae* has the genetic repertoire to adapt very well to many different hosts.

### 2.8. Streptococcus marimammalium

*S. marimammalium* was first isolated from the lung of a dead harbor seal and a dead grey seal in Iverness, Scotland [51]. In 2007/2008, it was also isolated (together with *S. agalactiae* and *S. canis)* from nasal and oral swabs of two healthy Hawaiian monk seals from the Waikiki Aquarium, Honolulu, Hawaii [68]. In 2016, it was also isolated from South American Fur Seal Pups with moderate to marked, multifocal, mucopurulent bronchopneumonia on Guafo Island, Chile, South America [98]. To our knowledge, nothing is known about virulence factors and pathogenicity of *S. marimammalium*. 

### 2.9. Streptococcus mitis

*S. mitis* is mainly known as member of the human oral cavity [134,135] and as opportunistic pathogen causing endocarditis and bloodstream infections in neutropenic and immunocompromised patients [136,137,138]. It is closely related to the human pathogen *S. pneumoniae* and its genome contain virulence genes involved in colonization and adherence, which might also be important for commensals to interact with host cells [139]. However, genes for hyaluronidase and capsular genes were absent. 

*S. mitis* was isolated in 1985 from a blowhole swab of a captive, healthy white whale (*Delphinapterus leucas*) 139 days after captivity at the Mystic Marinelife Aquarium Connecticut, USA [140]. In 1985, it was isolated from lesions of a grey seal with peritonitis in North Rona, Scotland [62] and between 2012–2014 from clinically normal eyes of two grey seals stranded on the British coast [67]. These findings suggest that *S. mitis* might also be a commensal in some marine mammals. The commensalism and pathogenesis of *S. mitis* is reviewed by Mitchel, 2011 [141].

### 2.10. Streptococcus phocae

*S. phocae* was first isolated and identified from lung, liver, spleen, and kidney samples of harbor seals suffering from pneumonia with areas of consolidation, purulent exudate in the bronchi, interlobular edema, and emphysema during a phocine distemper virus outbreak in northwestern Europe [34]. Later, *S. phocae* was also isolated from diseased Atlantic salmon [142,143], stranded southern sea otters [144], and as gut commensal of Indian white shrimp [145]. Two subspecies are described, *S. phocae* subsp. *salmonis* for isolates from Atlantic salmon and *S. phocae* subsp. *phocae* for isolates from seals [146]. 

In August 1994, beta-hemolytic streptococci with high similarity to *S. phocae* were isolated from spleen, liver, and kidney of Cape fur seals at Cape Cross, Namibia that suffered from respiratory infections and abortions [99]. A total of 69 *S. phocae* isolates were obtained from harbor and grey seals from the North and the Baltic Sea investigated between 1995 and 2000 [106]. A study on phocid seals (harbor and grey seals) that were older than 19 months from the North Sea of Schleswig-Holstein, Germany reported two *S. phocae* isolates from intestines of phocid seals with intestinal displacements [147]. During diagnostic evaluation by the Animal Health Center, Abbotsford, British Columbia, Canada *S. phocae* was isolated from harbor seals with an increase of prevalence since 2000, ringed seal (*P. hispida*) pups from arctic Canada and two stranded harbor porpoises from Washington State [48]. In spring and summer 2000, more than 10,000 Caspian seals (*Pusa caspica*) were found dead with canine distemper virus infection as primary diagnosis [47]. The investigated animals suffered from broncho-interstitial pneumonia, lymphocytic necrosis and depletion in lymphoid organs, and the presence of typical intracytoplasmic inclusion bodies in multiple epithelia. *S. phocae* was isolated from three of eight animals. Between 2001 and 2003, vaginal and preputial swabs of California Sea Lions were collected for investigations of genital bacterial infections and urogenital carcinoma [37]. *S. phocae* was isolated from three specimen of cancer and three specimens of non-cancer animals stranded along the central and northern California coast. In November 2007, a short-beaked common dolphin (*Delphinus delphis*) stranded at La Graciosa, Canary Islands [45]. Diagnostic evaluation revealed bacterial septicemia, fibrino-necrotizing to pyogranulomatous dermatitis and panniculitis, embolic pneumonia, neutrophilic and lymphoplasmacytic meningo-choroiditis, random neutrophilic hepatitis, lymphoplasmacytic myocarditis and epicarditis, necrotizing adrenalitis, suppurative endometritis, and multicentric reactive lymphadenopathy cutaneous purulent nodules in the tail fluke, vegetative mitral valve endocarditis, and presumed postpartum pyometra. *S. phocae* could be cultured from lung, brain, and adrenal gland tissue. Morbillivirus was detected in the epithelium of the choroid plexus of the fourth ventricle. In November 2009, a female spotted seal (*Phoca largha*) stranded at Kotzebue Sound, Alaska and was diagnosed with pyometra [148]. *S. phocae* was isolated from the purulent discharge in uterine contents. Three Navy bottlenose dolphins (*T. truncatus*) developed in the time between 2009 and 2010 a strangles-like syndrome associated with *S. phocae*, which was isolated after the animals showed clinical signs such as inflammatory hemogram, neutrophilic leukocytosis, and unilateral cervical lymphadenopathy [149]. Between 2004 to 2010 *S. phocae* could be isolated from five harbor seal pups of the Smith Island in Washington, USA that were also tested positive for phocine herpes virus [32]. *S. phocae* was also isolated from five cases of bacterial septicemia of white whales stranded in St. Lawrence Estuary between 1983 to 2012 [33]. Necropsy of a total of 241 harbor porpoises stranded at the eastern Pacific and western Atlantic coasts of Canada between 1988 to 2011 revealed bacterial septicemia with *S. phocae* isolation [150]. In winter 2012, an adult female Stellar sea lion stranded in South Korea and *S. phocae* was cultured from the liver [116]. The cause of death was unknown. During 85 postmortem investigations of marine mammals of the northeastern Pacific and arctic Canada stranded between 2007–2012 resulted in *S. phocae* isolates from harbor seals (*n* = 61), ringed seals (*n* = 5), harbor porpoises (*n* = 5), California sea lion (*n* = 7), Stellar sea lion (*n* = 3), Guadalupe fur seal (*Arctocephalus twonsendii*, *n* = 1) and elephant seal (*Mirounga angustirostris, n* = 1) [151]. Sequencing of 16S rRNA V4 hyper variable regions of blow samples collected from four wild healthy Indo-Pacific bottlenose dolphins (*T. aduncus*) in Shark Bay (SB), Western Australia, in 2012 identified *S. phocae* (and *S. equi*) [115]. In February 2014, *S. phocae* was isolated from a carcass of a subadult male northern seal at the coast of Niigata, Japan that suffered from necrotizing and fibrinous pneumonia with diffuse abscesses of all lung lobes and massive necrosis of kidney and liver [46]. Between 2010 to 2017 the health of captive and stranded Alaskan ice seals were investigated and *S. phocae* isolates were obtained from blood, abscess, and lymph node samples from ringed seals [152]. Harbor seals stranded at the coast of San Juan County, Washington, USA between 2002 to 2018 were examined and from one adult female animal a fatal septicemia caused by *S. phocae* was reported [153].

While the presence of an antiphagocytic capsule and virulence of *S. phocae* subsp. *salmonis* to Atlantic salmon has been demonstrated in infectivity experiments [142,154,155], whole genome analyses of *S. phocae* subsp. *phocae* identified typical streptococcal virulence factors such as fibronectin-binding proteins, the toxin streptolysin S and genes encoding for a capsule [156]. Invasion of fish and mammalian cell lines by *S. phocae* subsp. *phocae* has also been shown and confirmed its pathogenic potential [154]. 

However, *S. phocae* subsp. *phocae* also seems to be a commensal of the oral cavity of grey seals as revealed by microbiome analyses and 16S rRNA sequencing. A transmission of *S. phocae* to harbor porpoises via bites is also indicated [157] and *S. phocae* might be an opportunistic pathogen, at least for seals. 

### 2.11. Streptococcus viridans Group

In very few studies, streptococci isolated from marine mammals were identified as members of the *S. viridans* group (viridans streptococci), which includes streptococci that are usually alpha-hemolytic and inhabit the oral cavity, intestinal, and vaginal tract [158,159,160]. This group is very heterogeneous and includes species such as *S. anginosus*, *S. mitis*, *S. sanguinis*, *S. mutans,* and *S. salivarius*, which can also cause endocarditis [161], bacteremia [162], and respiratory infections [163].

Viridans streptococci were isolated from superficial abscesses, wounds, ocular and urethral discharges, and umbilici of live and from lung and liver samples of dead elephant seals, California sea lions and harbor seals stranded between January 1994 and December 1995 along the California Coast [164]. Viridans streptococci were isolated in mixed cultures with *Arcanobacterium phocae* from California sea lions, harbor seals, Northern elephant seals, sea otter and common dolphin stranded along the central California coast between 1994 and 2000 [165]. In Beluga whales that stranded at Cook Inlet (Alaska, USA) between 1998 and 2013 an isolate was identified as member of the *S. viridans* group [166]. Also, viridans streptococci were isolated from gastric fluid samples of free-ranging bottlenose dolphins from the southeastern United States during a catch and release health assessment between 2003 to 2005 [167]. 

### 2.12. One-Time Only Detections of Streptococcal Species from Marine Mammals

In studies described above, streptococcal species have been isolated and identified at least twice or more. In the following, reports on one-time only descriptions of streptococcal species are summarized. 

*S. uberis* was found in dead free-ranging male Antarctic fur seals with pneumonia and extensive polymorphonuclear infiltrations and necrosis or very widespread abscess formation and frequently there was an associated fibrinous exudative pleurisy [63]. *S. oralis* was isolated and identified by API strips from three swabs taken from healthy eyes of free-ranging grey seals stranded on the British coast between November 2012 and February 2014 [67]. In a metagenome dataset of blood, muscle, and fecal samples of a living stranded sperm whale (*Physeter catodon*) *S. anginosus*, *S. pneumoniae*, and *S. suis* were detected in blood and fecal samples, but not in the muscles [168]. The animal died 79 h after rescue. *S. intermedius* was detected in blow samples of free-ranging and presumably healthy grey whales from Magdalena Bay and the Gulf of California by polymerase chain reaction [169]. 

## 3. Streptococcal Infections in Marine Mammals: Virulence and Mechanims of Pathogenicity 

**Figure 2:**
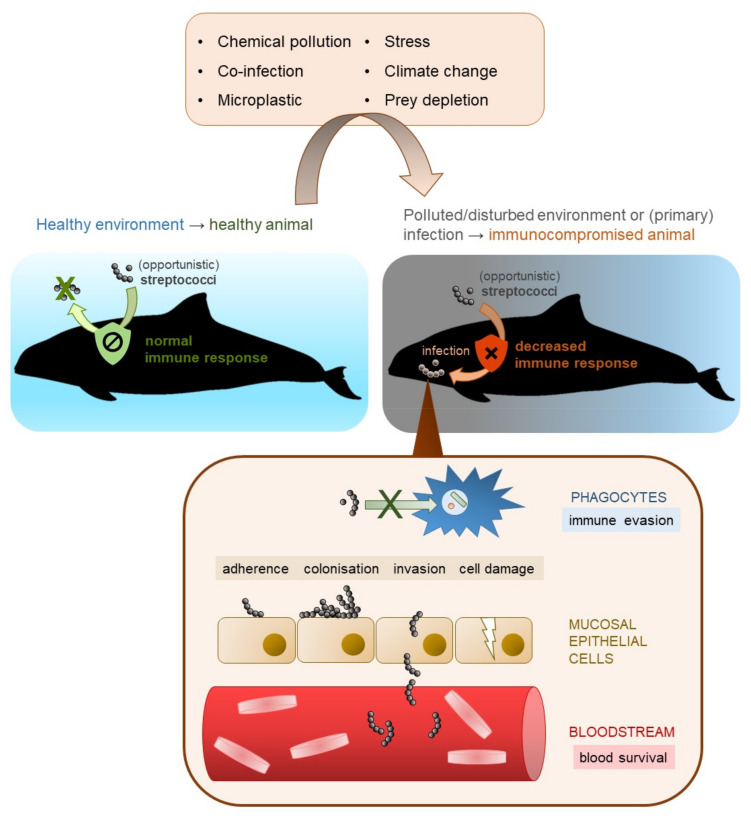
Schematic representation of the infection of marine mammals with (opportunistic) streptococci.

Streptococci are a phylogenetically diverse group and, hence, their virulence and infection mechanisms differ intensely. A general idea of streptococcal infection in (marine) mammals is displayed in Figure 2. One important requirement for a successful infection is the resistance of pathogenic streptococci to host phagocytosis. The major antiphagocytic factors are the polysaccharide capsule, which is also the basis for serotyping [127,170,171,172,173] and the streptococcal M protein of pyogenic streptococci [43,174]. The most critical phase in infection is the adhesion of streptococci to host cells. This is enabled by pili and/or adhesins such as fibronectin- and collagen-binding proteins and sortases [175,176]. It has also been demonstrated that streptococcal species are able to invade host cells [154,177,178] and produce toxins such as streptolysin S and streptolysin O [179,180]. There are numerous other virulence factors, for instance, streptokinases, secretory proteins that interacts with host plasminogen [181], or peptidoglycan deacetylases that protects the bacteria from host lysozymes [130,182]. To investigate the molecular mechanisms of streptococcal infection in marine mammals, primary cell cultures of marine mammals are required that can be infected and evaluated.

Many streptococci occur as opportunistic pathogens or as secondary infection [45,183,184,185,186,187]. This might also be the case for marine mammals. *S. agalactiae*, *S. canis* and *S. marimammalium* were found in the nasal cavity of two captured, healthy Hawaiian monk seals (*M. schauinslandi*) without any clinical signs [68]. A short-beaked common dolphin (*Delphinus delphis*) was coinfected by *Streptococcus phocae* and cetacean morbillivirus indicating *S. phocae* as secondary infection [45]. 16S rRNA sequencing of blow samples collected from four wild and healthy Indo-Pacific bottlenose dolphins (*Tursiops aduncus*) in Shark Bay, Western Australia identified *S. phocae*, *S. equi* and *Streptococcus* Group D as members of the blow microbiome without clinical signs [115]. 

Weakened or immunocompromised animals are more susceptible for infectious disease, even caused by opportunistic pathogens. The marine environment is threatened by climate change [16,17], ocean acidification [13,14,15], and pollutants (5,6,22,24), which can influence the immune system of marine mammals negatively [21,22,23,24]. This can result in an increase of infectious disease, which might also be true for streptococcal species [23]. 

The development of an infectious disease depends on the successful infection of a pathogen and on its virulence. This also includes the ability to evade host immune defense. Streptococci have evolved many mechanisms of immune invasion. For instance, the pore-forming toxin streptolysin O induces caspase-dependent macrophage apoptosis [188] and the gene sets for streptolysin S, which is responsible for the beta-hemolysis, was also found in *S. phocae* subsp. *phocae* [156]. The human pathogenic *S. pyogenes* can recruit and colonize collagen type IV via surface-bound fibronectin, and the collagen fibers protect the bacterial cells from opsonizing antibodies [189]. Proteins that interact with collagen were also found in other streptococcal species including *S. phocae* [190]. Group A streptococci (e.g., *S. pyogenes*) can bind red blood cells by S protein for immune evasion [191]. In *S. phocae* subsp. *phocae,* an immunoglobulin G degrading enzyme, called ideP, was identified, which cleaves IgG of seals and thus, contributes to immune evasion [192]. Hence, streptococci that infect marine mammals requires specific adaptation to their immune system, as only immune evasion guarantee virulence and infection. The immune system of marine mammals is similar to other (terrestrial) mammals regarding general mechanisms [193]. In grey seals, the main type of immunoglobulin was IgG with two subclasses [194]. Furthermore, the authors discussed whether the susceptibility for bacterial infections including streptococcal infections in grey seal pups is related to the observed low values of IgG in pup serum in comparison to the relatively high values in the colostrum. Immunoglobulin classes homologous to human IgG, IgM, and IgA were identified also in dolphins and sea lions [195] indicating that streptococci face similar conditions and molecules when jumping from terrestrial to marine mammals. 

One difference in streptococcal infection between marine and terrestrial mammals could be the fact that plasminogen from marine mammals could be activated by human plasma including urokinase, but not by streptokinase vaccine [196,197]. Moreover, it is known that the genes for streptokinase of group A streptococci showed a high heterogeneity even in strains with the same serotype indicating immunological and chemical differences [198]. We did not find any information on streptokinases in streptococci of marine mammals and, hence, it can only be speculated if they just lost the gene or if there a genetic variations reflecting host adaptability. 

## 4. Adaptation of Streptococci to Marine Mammals

Many streptococcal species found in marine mammals are also present in terrestrial mammals, although there are some physiological differences. That raises the question, to what extend and how these streptococci adapt to their specific marine host environments. Since this has not been studied yet in any detail here, we can only speculate and discuss it.

The body temperature of marine mammals is comparable to that of terrestrial mammals [199,200,201,202,203], thus, it does not require special adaptations. However, gas physiology and rapid change in hydrostatic pressure during diving might be a challenge for the bacteria. The hydrostatic pressure of seawater increases about 0.1 atmosphere per meter of depth. It has been shown that hydrostatic pressure can influence the growth and viability of marine and terrestrial bacteria negatively [204,205]. At least the closely related *Lactococcus lactis* (previously *S. lactis*) was able to survive 500 atmosphere representing around 5000 m depth, while many other bacteria including species of *Bacillus*, *Clostridium,* and *Staphylococcus* died at pressure of 400 or less atmospheres [205]. In conclusion, streptococcal species and other bacteria might not have a problem with the hydrostatic pressure that increases during diving of marine mammals. However, bacteria of marine mammals, depending on which body part they inhabit (e.g., skin, intestines, respiratory tract) and if they were acquired from the marine environment, have to challenge the salinity of seawater. Seawater has an average salinity of 3.5%, but salt tolerance of streptococcal species is usually tested at 6.5% NaCl and 40% bile salts, respectively, [34,142,206,207]. The salt tolerance of *S. iniae* was tested at 2.0%, 4.0%, and 6.5% NaCl and growth was observed for the first two conditions [122]. In addition, few *S. thermophilus* strains and *S. uberis* grow at 4.0% NaCl, but not at 6.5% [208,209]. The fish pathogen *S. parauberis* is known to persist in seawater, probably by switching to dormancy as a survival strategy [210]. Therefore, it is also possible for other streptococcal species to persist and maybe even grow in seawater. 

Streptococci infecting marine mammals often use the respiratory tract as port of entry for colonization infection. Thus, it is likely that they have adapted to this special host niche. Notably, the respiratory tract differs between terrestrial and marine mammals, e.g., in anatomy, immune response, gas physiology (O_2_/CO_2_ exchange), humidity, and chemical composition of the mucus. For instance, it has been shown that lung surfactant of pinnipeds has higher amounts of anti-adhesive components compared to terrestrial mammals, which probably supports alveolar opening after collapse during diving [211]. 

To our knowledge, there are no specific studies on genetic adaptation of streptococcal species to their marine mammalian host. However, whole genome analyses of human and fish/frog *S. agalactiae* strains revealed genetic adaptation to fish host by gene reduction and different gene expression such as of virulence associated genes [212]. A comparative genomic study of *S. dysgalactiae* species suggest that changes in gene content, selection of orthologous protein-coding loci and operon promoters involving mobile elements enables streptococci to adapt to changing environments and new hosts [213]. *S. phocae* subsp. *phocae* showed host specificity by the immunoglobulin G degrading enzyme ideP that solely cleaves IgG of grey and harbor seals, but not from harbor porpoises or non-marine mammals indicating functional adaptation [192]. *S. halichoeri* is assumed to have marine origin, although it has also been isolated from dog, bluefox, finnraccoon, and mink, as it has a great number of adhesins and salt tolerance proteins [120]. Lateral gene transfer between different streptococcal species is discussed as potential way of host adaptation [214]. For instance, lateral gene transfer was observed between *S. canis* and human *S. urinalis* and bovine *S. agalctiae* and *S, dysgalactiae* subsp. *dysgalactiae* mediated by variety of mobile genetic elements. This might also be true for marine mammals, where streptococci adapted to the marine mammalian host such as *S. phocae* might exchange genetic elements with acquired streptococcal species from fish or other sources. Further studies are required to understand the adaptation and interaction of streptococcal species and their marine mammal host as there is a huge scientific gap.

## 5. Epidemiology and Possible Transmission Routes of Streptococci Species in Marine Mammals

**Figure 3:**
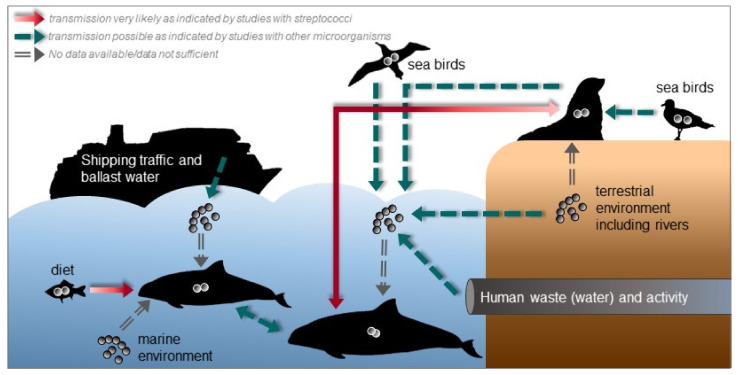
Possible ways how streptococci are introduced into marine environments and how they may be transmitted to and between marine mammals. Most indicated transmission routes are hypothetical based on general microbiological observations. In the other cases, there are studies showing that the transmission route is very likely or even confirmed. Hence, this figure displays scientific gaps that has still to be filled and where data are insufficient.

It is not known how marine mammals acquire or get infected with streptococci. Studies on the ecology and the environment could provide some insights about possible transmission routes and the epidemiology of streptococcal infections in marine mammals. In Figure 3, we give an overview about possible transmission routes and sources for streptococcal species. With many social species among marine mammals, transmission of pathogens are density- or frequency-dependent according to their social behavior [215,216]. 

The diet, e.g., fish could serve as reservoir for (pathogenic) streptococcal species and consequently as source of infection for marine mammals, which is also indicated by the study of Evans et al. [65], where the same *S. agalactiae* strain was found in both a dead dolphin and its diet, a mullet which was found in its stomach. *S. iniae* and *S. agalactiae* were also isolated from wild fish indicating fish as potential source for streptococci [217,218]. In addition, *S. halichoeri* is supposed to be transmitted to mink and Finnish dogs via fed fish [120]. *S. phocae* is also a well-known pathogen of Atlantic salmon [143]. However, the fish isolates differ from isolates of marine mammals, which was also the reason to suggest two subspecies of *S. phocae* [146]. The genetic differences could be a result of adaptation from fish to the mammalian host.

Pathogenic streptococci and other bacteria could be introduced into seawater by shipping traffic including ballast water [219,220], human activities including recreational activity [221,222,223,224,225] and waste (water) [66,226,227,228,229] and from the terrestrial environment via rivers and storm water [230,231], wind transport [232,233] or animals, e.g., semi-aquatic mammals, such as pinnipeds, from which streptococci are frequently isolated. For instance, human pathogenic *S. agalactiae* strains were identified in grey seals indicating that sea mammals were exposed to human pathogens via human effluents that contaminate coastal surface waters [66]. *S. phocae* has been found in the oral cavity of grey seals and in bite wounds of harbor porpoises probably caused by grey seals suggesting an interspecies transmission [157]. Seabirds can also shed pathogenic organisms into seawater [228,234,235] and streptococci were also detected in the gastrointestinal tract of seabirds [236]. If these transmission routes are real, streptococcal contamination and infections might increase with increasing human activities including growing cities in coastal regions and higher rates of shipping traffic, e.g., an increase of pollution and a higher likelihood of ship strikes or fisheries interactions. In addition, with higher temperatures due to climate change the persistence of pathogens in seawater is probably enhanced and, thus, there is a higher risk of infection [17,215]. The mortality of tilapias due to *S. agalactiae* infection was increased in higher water temperature [237]. This could also be the case for marine mammals, but there are no studies yet. In addition, habitat loss can enhance transmission by leading to higher population densities with higher contact rates.

To our knowledge streptococci are no or not abundant members of the natural marine microbiome [238]. This raises the question, where they come from. Nevertheless, streptococci that are introduced by the routes mentioned above might cause dermal disease or enter marine mammals through open wounds and other traumata. Díaz-Delgado et al. [45] proposed that the *S. phocae* infection in a short-beaked common dolphin occurred through cutaneous penetration after a skin traumata, as the dolphin showed cutaneous disease with firm, raised, and occasionally ulcerated purulent subcutaneous nodules along the ventral, dorsal, and cranial edge of the caudal fluke, bilaterally, and more prominently at its insertion with the peduncle.

However, studies on the distribution and presence of streptococci in healthy animals including the screening of the environment are necessary to investigate possible transmission routes or confirm these streptococcal species as commensals or members of the normal microbiome.

## 6. Conclusions and Outlook

Taken together, this is an overview about streptococcal species that were identified in marine mammals. Streptococcal species play an important role in the health of marine mammals all over the world. However, while beta-hemolytic streptococci are frequently isolated from marine mammals, only relatively few isolates were further identified (to the species level). There are further marine mammals from which (beta-hemolytic) streptococci have been isolated, but not identified to the species level, such as the blue whale (*Balaenoptera musculus*) [239], the gray whale (*Eschrichtius robustus*) [239], the sperm whale (*Physeter macrocephalus*) [240], the killer whale (*Orcinus orca*) [241], and the pacific walrus (*Odobenus rosmarus divergens*) [242]. This scientific gap makes it difficult to evaluate the diversity, distribution, and epidemiology of streptococcal species among marine mammals and needs to be filled. In addition, while there are lineages of streptococcal species that are quite host-specific, there are others that seem to have a more groad host spectrum and are easily transmissible between different hosts. For instance, in addition to marine mammals, *S. canis* can be found in dogs and cows [86], *S. halichoeri* in humans [119], and *S. phocae* in Atlantic salmon [143] and shrimps [145]. For some of these species, subspecies were defined based on their host-related differences such as *S. phocae* subsp. *phocae* and *S. phocae* subsp. *salmonis* [146] or *S. halichoeri* subsp. *halichoeri* and *S. halichoeri* subsp. *hominis* [119]. Furthermore, some of the streptococcal species found in marine mammals are major fish pathogens such as *S. agalactiae* [243], *S. iniae* [244], and *S. phocae* [143] and even zoonotic infections are possible, but the lack of data does not allow clear risk assessments. *S. iniae* caused infections in humans that handled live or freshly killed fish [58,245]. *S. canis* infections in humans are summarized by Galpérine et al. [56] and an endocarditis due to *S. canis* has been reported by Ansallem et al. [246]. *S. equi* subsp. *zooepidemicus* is also known to cause zoonotic disease in humans [53,247]. Hence, people working with marine mammals should also be aware of the zoonotic potential of streptococcal species.

It is also not fully understood how the different streptococcal species are involved in diseases of marine mammals, although they are frequently isolated from sick or dead animals. With increasing chemical pollution and other anthropogenic activities in the marine ecosystem, the health of marine mammals is threatened and thus, they are more susceptible to infectious diseases. Hence, more research is needed on the epidemiology and pathogenic potential of streptococcal species in marine mammals.

In conclusion, streptococcal species are isolated from many different marine mammal species world-wide. Further investigations on the role of the different streptococci species on the health status of marine mammals is urgently needed as streptococci are found with high prevalence in diseased marine mammals. This also underlines the need of additional information on the zoonotic potential of streptococci species found in marine mammals.

## Data Availability

Not applicable.

## Supplementary Materials

The following is available online at https://www.mdpi.com/2076-2607/9/2/350/s1, Table S1: Prevalence of isolated and identified streptococcal species in marine mammals.
