# Supplementary material for: Streptococcal Infections in Marine Mammals
Source: Microorganisms. 2021 Feb 10;9(2):350. doi: 10.3390/microorganisms9020350 (PMC7916692; doi:10.3390/microorganisms9020350)
Supplement: Supplementary file 1 [file microorganisms-09-00350-s001.pdf]

Supplementary table 1: Prevalence of isolated and identified streptococcal species in marine mammmals (only findings that occur more than once are shown).

	Carnivora															*Cetacea*							
	Pinnipedia															*Mysticeti*	*Odontoceti*						
	Phocidae								*Otariidae*							*Balaenidae*	*Phocoenidae*	*Delphinidae*				*Iniidae*	*Monodontidae*
	Monachus schauinslandi	*Monachus monachus*	*Phoca vitulina*	*Phoca largha*	*Halichoerus grypus*	*Pusa caspica*	*Pusa hispida*	*Mirounga angustirostris*	*Arctocephalus australis*	*Arctocephalus gazella*	*Arctocephalus pusillus*	*Arctocephalus twonsendii*	*Zalophus californianus*	*Eumetopias jubatus*	*Callorhinus ursinus*	*Eubalaena australis*	*Phocoena phocoena*	*Globicephala melaena*	*Tursiops truncatus*	*Tursiops aduncus*	*Delphinus delphis*	*Inia geoffrensis*	*Delphinapterus leucas*
S. agalactiae	x				x					x									x				
S. bovis		x	x							x									x				
S. canis	x		x						x		x		x		x		x						
S. dysgalactiae		x			x											x	x						
S. equi			x		x					x	x						x	x	x	x			
S. halichoeri					x									x									
S. iniae																			x			x	
S. marimammalium	x		x		x				x														
S. mitis					x																		x
S. phocae			x	x	x	x	x	x			x	x	x	x	x		x		x	x	x		x
